# A new species of *Rana* (Amphibia, Anura, Ranidae) from Hainan, Southern China

**DOI:** 10.3897/zookeys.1296.211503

**Published:** 2026-11-04

**Authors:** Hao-Tian Wang, Ming-Hong Huang, Chen-Xi Liao, Xian-Chun Qiu, Han-Ming Song, Shuo Qi, Da-Er Ding, Shang-Biao Zhuo, Wei Wang, Jun Wu, Bin Wang, Ying-Yong Wang, Pi-Peng Li, Ji-Chao Wang

**Affiliations:** 1 Ministry of Education Key Laboratory for Ecology of Tropical Islands, Key Laboratory of Tropical Animal and Plant Ecology of Hainan Province, College of Life Sciences, Hainan Normal University, Haikou 571158, China Ministry of Education Key Laboratory for Ecology of Tropical Islands, Key Laboratory of Tropical Animal and Plant Ecology of Hainan Province, College of Life Sciences, Hainan Normal University Haikou China; 2 Institute of Herpetology, Shenyang Normal University, Shenyang 110034, China Institute of Herpetology, Shenyang Normal University Shenyang China; 3 Hainan Shangxi Provincial Nature Reserve, Wanning 571500, Hainan, China Hainan Shangxi Provincial Nature Reserve Wanning China; 4 Independent Researcher, Haikou 571100, China Unaffiliated Haikou China; 5 School of Ecology, Sun Yat-sen University, Shenzhen 518107, Guangdong, China School of Ecology, Sun Yat-sen University Guangdong China; 6 School of Science, Qiongtai Normal University, Haikou 571127, China School of Science, Qiongtai Normal University Haikou China; 7 Nanjing Institute of Environmental Sciences, Ministry of Ecology and Environment of China, Nanjing 210042, China Nanjing Institute of Environmental Sciences, Ministry of Ecology and Environment of China Nanjing China; 8 Chengdu Institute of Biology, Chinese Academy of Sciences, Chengdu 610041, China Chengdu Institute of Biology, Chinese Academy of Sciences Chengdu China; 9 School of Life Sciences, Sun Yat-sen University, Guangzhou 510275, China School of Life Sciences, Sun Yat-sen University Guangzhou China

**Keywords:** Hainan Island, historical records, integrative taxonomy, molecular phylogeny, *Rana johnsi* group

## Abstract

A new species of the genus *Rana* is described from Hainan Island, southern China, based on morphological and molecular evidence. During herpetological surveys conducted in Wanning City and Mt. Diaoluo, Hainan Province, a series of *Rana* specimens assignable to the *R. johnsi* group were collected from montane tropical rainforest habitats. Phylogenetic analyses based on mitochondrial 16S, COI, and cyt *b* sequences recovered the Hainan population as a distinct and well-supported lineage within the *R. johnsi* group. Morphometric analyses, including PCA, DAPC, and PERMANOVA, further demonstrated significant differentiation between the Hainan population and its congeners. The new species, *Rana hainan* Wang, Qiu, Song, Qi, Wang, Li & Wang, **sp. nov**., is characterized by a medium body size, head longer than wide, a distinct triangular black patch on the temporal region, straight and narrow dorsolateral folds, toe tips with connected lateroventral grooves, absence of tarsal folds, and breeding males possessing a continuous grayish-white nuptial pad on finger I. Historical records of *R. varians*, *R. nigrotympanica*, and *R. johnsi* from Hainan are reviewed. Records from Mt. Diaoluo are reassigned to the new species, whereas records from other localities are conservatively treated as *Rana cf. johnsi* pending further investigation.

## Introduction

*Rana* Linnaeus, 1758, represents a taxonomically complex and widely distributed lineage within the family Ranidae, occurring broadly across temperate regions of the Northern Hemisphere, ranging from temperate Eurasia through Indochina and extending into western North America from Alaska to northern Baja California, Mexico (Frost 2026). Traditionally recognized as one of the most speciose genera of amphibians, *Rana* sensu lato has undergone substantial taxonomic revision over the past several decades, driven by both morphological reassessment and advances in molecular phylogenetics. Early comprehensive treatments (e.g., Boulenger 1920; Dubois 1981, 1992) proposed detailed infrageneric classifications; however, many of these arrangements have since been challenged or revised due to issues such as poorly defined diagnostic characters and non-monophyletic groupings (Inger 1996; Frost et al. 2006). Subsequent phylogenetic studies employing mitochondrial and nuclear DNA data have greatly improved resolution within the genus, supporting the monophyly of *Rana* sensu lato while also highlighting deep evolutionary divergences among major lineages (Macey et al. 2001; Wiens et al. 2009; Pyron and Wiens 2011; Yuan et al. 2016). Despite these advances, the taxonomic status of *Rana* remains contentious. Some authors advocate retaining a broad concept of *Rana* comprising multiple subgenera (e.g., *Rana*, *Amerana*, *Aquarana*, *Lithobates*, *Liuhurana*, *Pantherana*, *Pseudorana*, and *Zweifelia*; Yuan et al. 2016), whereas others propose elevating these subgenera to full generic rank, thereby restricting *Rana* sensu stricto to a smaller number of species (Dubois et al. 2021; Frost 2026).

In China, *Rana* sensu lato is currently represented by approximately 30 recognized species (AmphibiaChina 2026). Among these species, those assigned to the subgenus *Rana* are further classified into species groups. Based on morphological evidence, Fei et al. (2009) recognized three species groups: the *R. longicrus* group, the *R. chensinensis* group, and the *R. amurensis* group. Subsequent phylogenetic studies have refined the composition and boundaries of these groups (Yan et al. 2011; Zhou et al. 2015, 2017; Yuan et al. 2016; Wang et al. 2017; Zhao et al. 2017), and the former *R. longicrus* group has been redefined as the *R. japonica* group (Yang et al. 2017). More recently, Wan et al. (2020) established the *R. johnsi* group based on molecular phylogenetic evidence. As a result, four species groups: the *R. amurensis* group, *R. chensinensis* group, *R. japonica* group, and *R. johnsi* group, are currently recognized within the subgenus *Rana* in China. Nevertheless, certain taxa (e.g., *R. arvalis* Nilsson, 1842 and *R. asiatica* Bedriaga, 1898) remain of uncertain placement, highlighting ongoing difficulties in resolving intrageneric relationships. Moreover, extensive morphological homoplasy and the presence of cryptic diversity have historically obscured species boundaries within the genus, contributing to persistent taxonomic uncertainty (Dubois 1992; Frost et al. 2006; Che et al. 2007).

Previous studies have reported Hainan populations of *Rana* under different taxonomic names. Liu et al. (1973) first reported the occurrence of *Rana varians* Boulenger, 1894 in Mt. Jianfengling, Hainan, but only a single poorly preserved specimen was available, and a question mark was placed after the species name to indicate uncertainty in identification. Later, Dubois (1992) revised the Chinese populations of *R. varians* to R. (Sylvirana) nigrotympanica Dubois, 1992, and subsequent studies followed this taxonomic treatment (Wang et al. 2007; Fei et al. 2009; Shi et al. 2011). Fei et al. (2005) expanded the known distribution of this taxon in Hainan to include Mt. Jianfengling, Danzhou, Dongfang, Baisha, Tunchang, Qiongzhong, Mt. Diaoluo, and Mt. Wuzhi; Wang et al. (2007) confirmed its occurrence in Hainan based on newly collected specimens from Mt. Diaoluo. Subsequently, Fei et al. (2009) added *R. johnsi* Smith, 1921 to the Hainan herpetofauna (Mt. Jianfeng), and Shi et al. (2011), based on the same batch of Mt. Diaoluo specimens as Wang et al. (2007) (sharing identical collection dates and measurement data), reassigned the Mt. Diaoluo population to *R. johnsi* and added a distribution record for Mt. Yinggeling. At the same time, they retained records of *R. nigrotympanica* from multiple localities in Hainan and noted that its taxonomy and distribution required further study.

During recent field surveys in Wanning City and Mt. Diaoluo, Hainan Province, China, several specimens of *Rana* were collected. Detailed morphological examination indicates that these specimens belong to the *Rana johnsi* group of the subgenus *Rana*, as evidenced by the presence of lateroventral grooves on the flattened tips of the toes and a distinct triangular black patch on the temporal region. The specimens, however, are readily distinguishable from all currently recognized congeners. Phylogenetic analyses further corroborate their distinctiveness, recovering them as an independent evolutionary lineage within the genus. Based on the combined morphological and molecular evidence, we herein describe this population from Wanning and Mt. Diaoluo, Hainan, as a new species of the subgenus *Rana*.

## Materials and methods

### Sampling

**Figure 1.**
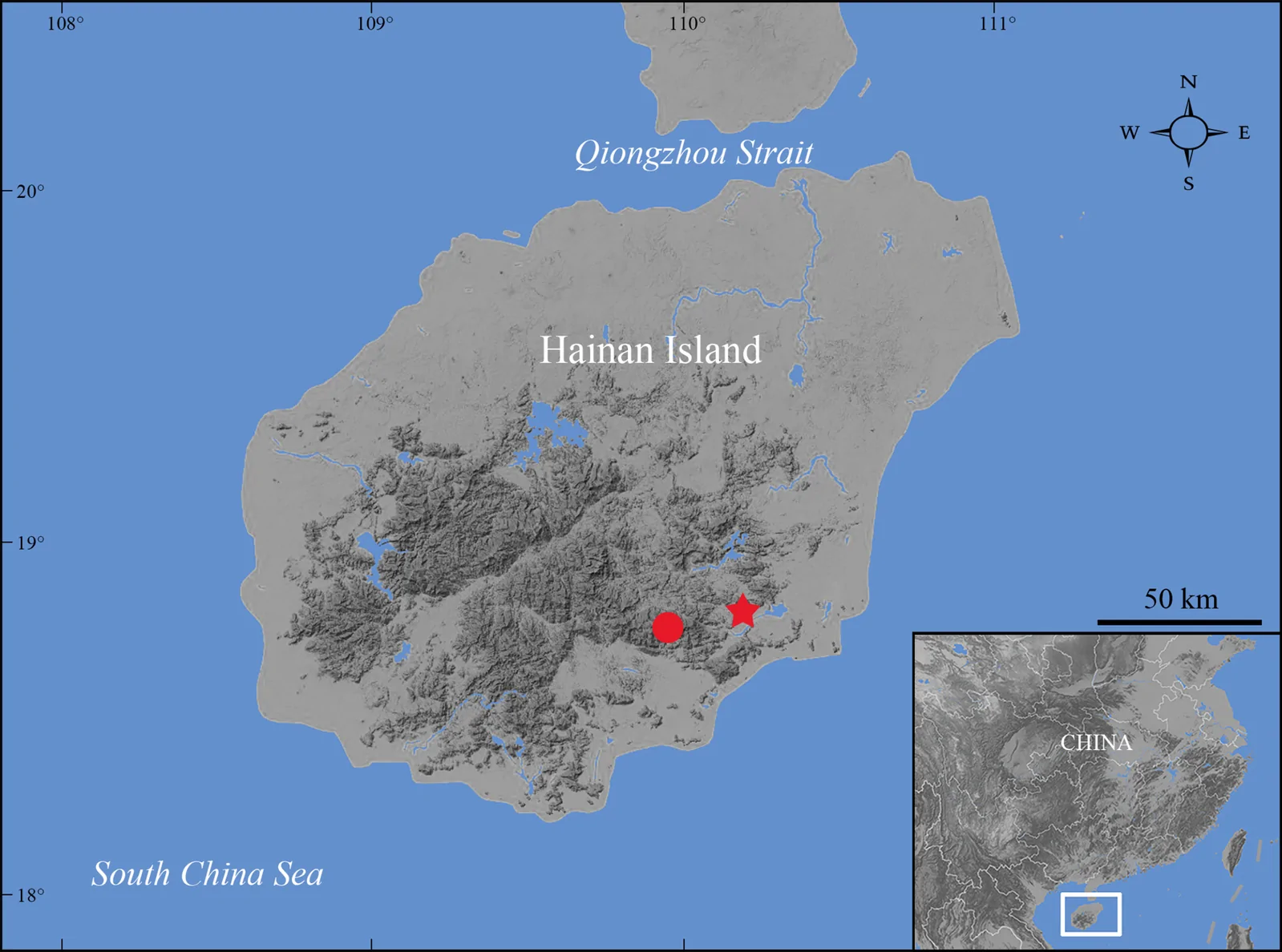
Map showing the sampling localities of *Rana hainan* sp. nov. on Hainan Island. Red pentagram: Shangxi Provincial Nature Reserve, Wanning, Hainan, China; red circle: Mt. Diaoluo, Lingshui, Hainan, China.

Twelve specimens of the subgenus *Rana* were collected from Shangxi Provincial Nature Reserve, Wanning City, and Mt. Diaoluo, Lingshui County, Hainan Province, China (Fig. 1) from January 2024 to April 2026. After capture, these frogs were photographed, and then euthanized using tricaine methanesulfonate (MS-222), fixed in 4% buffered methanal, and preserved in 75% ethanol at about 25 °C. Thigh muscle and liver tissues were excised before fixation for molecular analyses and preserved in absolute ethanol at –40 °C. Specimens were deposited at Hainan Normal University (HNU), Shenyang Normal University (SYNU) and Sun Yat-sen University (SYS).

### Morphology

The morphological terminologies and measurements followed Wu et al. (2021), with the following morphometric characters being used: snout-vent length (**SVL**, distance from the tip of the snout to the posterior edge of the vent); head length (**HDL**, distance from the tip of the snout to the articulation of jaw); maximum head width (**HDW**, greatest width between the left and right articulations of jaw); snout length (**SL**, distance from the tip of the snout to the anterior corner of the eye); internasal distance (**IND**, minimum distance between the inner margins of the external nares); interorbital distance (**IOD**, minimum distance between the inner edges of the upper eyelids); eye diameter (**ED**, distance from the anterior corner to the posterior corner of the eye); maximal tympanum diameter(**TYD**); upper eyelid width (**UEW**, greatest width of the upper eyelid margins measured perpendicular to the anterior-posterior axis); hand length (**HAL**, distance from tip of third digit to proximal edge of inner palmar tubercle); length of lower arm and hand (**LAL**, distance from the elbow to the distal end of the Finger IV); lower arm width (**LW**, maximum width of the lower arm); foot length (**FL**, distance from tarsus to the tip of fourth toe); length of foot and tarsus (**TFL**, distance from the tibiotarsal articulation to the distal end of the Toe IV); thigh length (**THL**, distance from vent to knee); tibia length (**TL**, distance from knee to tarsus); maximal tibia width (**TW**). Measurements were conducted on fixed specimens with digital calipers (Deli DL91200 Digital Vernier Caliper) to the nearest 0.1 mm. Sex was determined based on secondary sexual characteristics (nuptial pads and nuptial spines) and by gonadal examination. Additional morphological data of the *Rana johnsi* group species were obtained from the literature (Smith 1921; Shen 1986; Fei et al. 2009; Wu et al. 2021).

### Statistical analyses

Only male specimens were included in the statistical analyses due to the limited availability of females. The dataset comprised three individuals from Hainan, 10 of *R. johnsi*, 27 of *R. sangzhiensis* Shen, 1986, and four of *R. wuyiensis* Wu, Shi, Zhang, Chen, Cai, Hoang, Wu & Wang, 2021.

All morphometric variables were natural log-transformed (ln) to improve normality and reduce heteroscedasticity. To remove allometric effects of body size using the following equation: X_a_ = X_ln_ – β • (SVL_ln_ – SVL_m_), where X_a_ = adjusted value; X_ln_ = ln-transformed measurements; β = unstandardized regression coefficient for each species; SVL_ln_ = ln-transformed SVL; and SVL_m_ = overall average SVL_ln_ of all samples (Thorpe 1975; Chan and Grismer 2022). Univariate analyses were performed on size-adjusted variables to assess differences among groups. For each variable, assumptions of normality and homogeneity of variances were evaluated using the Shapiro–Wilk and Levene’s tests, respectively. Depending on whether assumptions were met, either one-way ANOVA or the Kruskal–Wallis test was applied. When significant, post hoc pairwise comparisons were performed using Tukey’s HSD or Dunn’s test, respectively, with Bonferroni-adjusted p-values. Principal Component Analysis (PCA) was conducted on the size-adjusted and standardized dataset to summarize patterns of morphological variation and to visualize group differentiation in multivariate space. Discriminant Analysis of Principal Components (DAPC) was subsequently performed to further assess morphological differentiation among predefined groups and maximize between-group variation while minimizing within-group variation (Grismer et al. 2022). To further test for overall morphological differences among groups, a permutational multivariate analysis of variance (PERMANOVA) was performed based on Euclidean distances derived from the PCA input data. Statistical significance was assessed using 10,000 permutations. All analyses and visualization were conducted in R v. 4.4.2 (R Core Team 2024) using the package *GroupStruct2* (Chan and Grismer 2025).

### Molecular phylogenetic analyses

Genomic DNA was extracted from tissue samples using a DNA extraction kit TIANamp Genomic DNA Kit from Tiangen Biotech (Beijing) Co., Ltd. Three mitochondrial DNA fragments were amplified: the partial 16S ribosomal RNA coding sequence (16S), the partial cytochrome c oxidase subunit 1 gene (COI) and the partial cytochrome b (cyt *b*) gene. PCR primers and reaction conditions are provided in Table 1. The PCR products were purified using spin columns and sequenced with forward primers on an ABI 3730 automated genetic analyzer by Tianyi Huiyuan Biotech (Wuhan) Co., Ltd. These newly obtained sequences were uploaded to GenBank (Table 2).

**Table 1.** Primers and annealing temperatures used in this study.

**Gene**	**Primer**	**Sequence**	**Annealing temperature (°C)**	**Reference**
16S	L3975	5'-CGCCTGTTTACCAAAAACAT-3'	51	Simon et al. (1994)
	H4551	5'-CCGGTCTGAACTCAGATCACGT-3'		
	L2A	5'-CCAAACGAGCCTAGTGATAGCTGGTT-3'		Chen et al. (2013)
	H10	5'-TGATTACGCTACCTTTGCACGGT-3'		
COI	dgLCO	5'-GGTCAACAAATCATAAAGAYATYGG-3'	45	Meyer et al. (2005)
	dgHCO	5'-AAACTTCAGGGTGACCAAARAAYCA-3'		
cyt *b*	HERP328	5'-GAAAARCTRTCGTTGTWATTCAACTA-3'	52	Yuan et al. (2016)
	HERP329	5'-CTACKGGTTGTCCYCCRATTCATGT-3'	53	

**Table 2.** Localities, voucher information, and GenBank accession numbers for all samples used in this study. “/” unavailable data.

**No**.	**Species**	**Voucher ID**	**Locality**	**16S**	**COI**	**cyt *b***
1	*Rana hainan* sp. nov.	SYS a009526	China: Hainan: Wanning: Shangxi Nature Reserve	QB070440	QB070455	QB074718
2	*Rana hainan* sp. nov.	SYS a009527**/**SYNU 900774	China: Hainan: Wanning: Shangxi Nature Reserve	QB070441	QB070456	QB074719
3	*Rana hainan* sp. nov.	SYS a009638	China: Hainan: Wanning: Shangxi Nature Reserve	QB070442	QB070457	QB074720
4	*Rana hainan* sp. nov.	SYS a009746	China: Hainan: Mt. Diaoluo	QB070443	QB070458	QB074721
5	*Rana hainan* sp. nov.	HNU 260407	China: Hainan: Mt. Diaoluo	QB070444	QB070459	QB074722
6	*Rana hainan* sp. nov.	HNU 260408	China: Hainan: Mt. Diaoluo	QB070445	QB070460	QB074723
7	*Rana hainan* sp. nov.	HNU 260409	China: Hainan: Mt. Diaoluo	QB070446	QB070461	QB074724
8	*Rana hainan* sp. nov.	HNU 260411	China: Hainan: Wanning: Shangxi Nature Reserve	QB070447	QB070462	QB074725
9	*Rana hainan* sp. nov.	HNU 260412	China: Hainan: Wanning: Shangxi Nature Reserve	QB070448	QB070463	QB074726
10	*Rana hainan* sp. nov.	HNU 260413	China: Hainan: Wanning: Shangxi Nature Reserve	QB070449	QB070464	QB074727
11	*Rana hainan* sp. nov.	HNU 260414	China: Hainan: Wanning: Shangxi Nature Reserve	QB070450	QB070465	QB074728
12	*Rana wuyiensis*	LSU202109001WYS01	China: Fujian: Wuyi Mountain	OL467321	OL467321	OL467321
13	*Rana wuyiensis*	CIB WY20201106016	China: Fujian: Wuyi Mountain	MZ337980	/	MZ355497
14	*Rana wuyiensis*	CIB WY20201106018	China: Fujian: Wuyi Mountain	MZ337981	/	MZ355498
15	*Rana wuyiensis*	CIB WY20201106007	China: Fujian: Wuyi Mountain	MZ337982	/	MZ355499
16	*Rana wuyiensis*	CIB WY20201106005	China: Fujian: Wuyi Mountain	MZ337983	/	MZ355500
17	*Rana wuyiensis*	CIB WY20200913003	China: Fujian: Wuyi Mountain	MZ337984	/	MZ355501
18	*Rana wuyiensis*	CIB WY20200913001	China: Fujian: Wuyi Mountain	MZ337985	/	MZ355502
19	*Rana wuyiensis*	CIB WY20201106017	China: Fujian: Wuyi Mountain	MZ337986	/	MZ355503
20	*Rana wuyiensis*	CIB WY20201106006	China: Fujian: Wuyi Mountain	MZ337987	/	MZ355504
21	*Rana wuyiensis*	CIB WY20200913002	China: Fujian: Wuyi Mountain	MZ337988	/	MZ355505
22	*Rana wuyiensis*	CIB WYS20200829003	China: Fujian: Wuyi Mountain	MZ337989	/	MZ355506
23	*Rana wuyiensis*	CIB WYS20200829002	China: Fujian: Wuyi Mountain	MZ337990	/	MZ355507
24	*Rana wuyiensis*	CIB WYS20200829001	China: Fujian: Wuyi Mountain	MZ337991	/	MZ355508
25	*Rana sangzhiensis*	CIB GL150097	China: Sichuan: Gulin	MZ337992	/	MZ355509
26	*Rana sangzhiensis*	CIB GL150091	China: Sichuan: Gulin	MZ337993	/	MZ355510
27	*Rana sangzhiensis*	CIB GL150088	China: Sichuan: Gulin	MZ337994	/	MZ355511
28	*Rana sangzhiensis*	CIB GL150011	China: Sichuan: Gulin	MZ337995	/	MZ355512
29	*Rana sangzhiensis*	CIB GL150010	China: Sichuan: Gulin	MZ337996	/	MZ355513
30	*Rana sangzhiensis*	CIB GL150068	China: Sichuan: Gulin	MZ337997	/	MZ355514
31	*Rana sangzhiensis*	SYS a004286	China: Hunan: Sangzhi: Tianping Mountain	MT408972	MT418634	/
32	*Rana sangzhiensis*	CIB SZ2012062103	China: Hunan: Sangzhi: Tianping Mountain	MZ337998	/	/
33	*Rana sangzhiensis*	CIB SZ2012062106	China: Hunan: Sangzhi: Tianping Mountain	MZ337999	/	/
34	*Rana sangzhiensis*	CIB SZ2012062104	China: Hunan: Sangzhi: Tianping Mountain	MZ338000	/	MZ355515
35	*Rana sangzhiensis*	CIB TPS20190413-FTY1-5	China: Hunan: Sangzhi: Tianping Mountain	MZ338001	/	MZ355516
36	*Rana sangzhiensis*	CIB TP20190413-36	China: Hunan: Sangzhi: Tianping Mountain	MZ338002	/	MZ355517
37	*Rana sangzhiensis*	CIB TPS20190413-FTY1-1	China: Hunan: Sangzhi: Tianping Mountain	MZ338003	/	MZ355518
38	*Rana sangzhiensis*	CIB TPS20190413-FTY1-2	China: Hunan: Sangzhi: Tianping Mountain	MZ338004	/	MZ355519
39	*Rana sangzhiensis*	CIB TPS20190413-FTY1-3	China: Hunan: Sangzhi: Tianping Mountain	MZ338005	/	MZ355520
40	*Rana sangzhiensis*	CIB TPS20190413-FTY1-4	China: Hunan: Sangzhi: Tianping Mountain	MZ338006	/	MZ355521
41	*Rana sangzhiensis*	SCUM 0405190CJ	China: Sichuan: Hongya: Zhangcun	KX269206	MF149929	KX269352
42	*Rana sangzhiensis*	CIB SZ2012062102	China: Hunan: Sangzhi: Tianping Mountain	MZ338007	/	MZ355522
43	*Rana johnsi*	ABV-00203	Vietnam: Lam Dong: Loc Bao	KX269182	/	KX269328
44	*Rana johnsi*	NNU 1910030	China: Guangxi: Shiwandashan Mountains	MZ338008	/	MZ355523
45	*Rana johnsi*	NNU 1910023	China: Guangxi: Shiwandashan Mountains	MZ338009	/	MZ355524
46	*Rana johnsi*	NNU 1910010	China: Guangxi: Shiwandashan Mountains	MZ338010	/	MZ355525
47	*Rana johnsi*	NNU 1910032	China: Guangxi: Shiwandashan Mountains	MZ338011	/	MZ355526
48	*Rana johnsi*	NNU 1910009	China: Guangxi: Shiwandashan Mountains	MZ338012	/	MZ355527
49	*Rana johnsi*	NNU 1910001	China: Guangxi: Shiwandashan Mountains	MZ338013	/	MZ355528
50	*Rana johnsi*	NNU 1910035	China: Guangxi: Shiwandashan Mountains	MZ338014	/	MZ355529
51	*Rana johnsi*	NNU 1910021	China: Guangxi: Shiwandashan Mountains	MZ338015	/	MZ355530
52	*Rana johnsi*	NNU 1910017	China: Guangxi: Shiwandashan Mountains	MZ338016	/	MZ355531
53	*Rana johnsi*	NNU 1910002	China: Guangxi: Shiwandashan Mountains	MZ338017	/	MZ355532
54	*Rana johnsi*	GXJX20180723-1	China: Guangxi: Jinxiu	MZ571365	MZ571365	MZ571365
55	*Rana johnsi*	CIB 20070712-1	China: Guangxi: Jinxiu	MZ338018	/	MZ355533
56	*Rana johnsi*	CIB 20070712	China: Guangxi: Jinxiu	MZ338019	/	MZ355534
57	*Rana johnsi*	IEBR.A 4849	Vietnam: Cao Bang	MZ338020	/	MZ355535
58	*Rana johnsi*	IEBR.A 4848	Vietnam: Cao Bang	MZ338021	/	MZ355536
59	*Rana johnsi*	CIB 201204008	Vietnam: Cao Bang	MZ338022	/	MZ355537
60	*Rana amurensis*	MSUZP-SLK-RUS49	Russia: Tomskaya: Teguldetskii	KX269203	/	KX269349
61	*Rana arvalis*	MSUZP-SLK-MKR21	Russia: Mordovia: Chamzinskii	KX269197	/	KX269344
62	*Rana asiatica*	KIZ-XJ0251	China: Xinjiang: 47tuan	KX269200	PQ434917	KX269346
63	*Rana chaochiaoensis*	SCUM 0405170CJ	China: Sichuan: Zhaojue	KX269192	/	KX269339
64	*Rana chensinensis*	KIZ RD05SHX01	China: Shaanxi: Huxian	KX269186	JF939080	KX269333
65	*Rana coreana*	MMS 223	South Korea	KX269202	MF149928	KX269348
66	*Rana culaiensis*	KIZ SD080501	China: Shandong: Culai Mountain	KX269190	JF939082	KX269337
67	*Rana dabieshanensis*	AHU 2016R001	China: Anhui: Dabie Mountains	MF172963	/	MF172964
68	*Rana dalmatina*	MSUZP-NPUA-R-21-1	Ukraine: Zakarpatska: Uzhgorod	KX269198	/	/
69	*Rana dybowskii*	MSUZP-IVM-1d	Russia: Primorye: Khasanskii	KX269188	/	KX269335
70	*Rana graeca*	ZMMU A-4293-1	Crna Gora (Montenegro): Niksic environs	KX269199	/	KX269345
71	*Rana hanluica*	KIZ GX07112915	China: Guangxi: Maoer Mountain	KX269191	/	KX269338
72	*Rana huanrensis*	SYNU 040006	China: Liaoning: Huanren	/	JF939072	JN984545
73	*Rana iberica*	ZMMU A-4292-1	Portugal: Porto: Valongo environs	KX269195	/	KX269342
74	*Rana japonica*	KIZ-YPX11775	Japan: Isumi-shi: Chiba Prefecture	KX269220	JF939101	KX269364
75	*Rana jiemuxiensis*	KIZ 05263	China: Hunan: Jiemuxi	/	JF939090	JF939127
76	*Rana jiulingensis*	SYS a005519	China: Jiangxi: Guanshan	MT408985	MT418647	/
77	*Rana kobai*	KUHE: 10051	Japan: Amami	AB685778	/	/
78	*Rana kukunoris*	KIZ CJ06102001	China: Qinghai: Qinghai Lake	KX269185	JF939073	KX269332
79	*Rana latastei*	Veith 2003	Italy: Campagna: Seseglio 2 km	AY147946	/	AY147967
80	*Rana longicrus*	NMNS 15022	China: Taiwan: Miaoli: Xiangtianhu	KX269189	/	KX269336
81	*Rana luanchuanensis*	KIZ047393	China: Henan: Luanchuan	/	MF149920	/
82	*Rana macrocnemis*	MSUZP-LFM-12	Russia: Daghestan: Agulskiy	KX269194	/	KX269341
83	*Rana maoershanensis*	SYNU 08030061	China: Guangxi: Mt Maoershan	KX139722	KX139728	KX139731
84	*Rana minuscula*	SWU 0012282	China: Zhejiang: Lishui	PQ350269	/	/
85	*Rana omeimontis*	SCUM 0405196CJ	China: Sichuan: Hongya	KX269193	/	KX269340
86	*Rana ornativentris*	KIZ-JP080101	Japan: Kyoto	KX269187	/	KX269334
87	*Rana pirica*	MSUZP-NPFE-R-08-42	Russia: Sakhalinskaya: Makorovskiy	KX269184	/	KX269331
88	*Rana pyrenaica*	ZFMK 65447-65448	Spain: Zuriza: Aragón	AY147950	/	AY147971
89	*Rana qui*	HNNU2302I026	China: Henan: Nanzhao	PQ881622	/	/
90	*Rana sakuraii*	KIZJP080104	Japan: Tokyo	KX269205	/	KX269351
91	*Rana sauteri*	SCUM 0405175CJ	China: Taiwan: Kaohsiung	KX269204	/	KX269350
92	*Rana shuchinae*	CIB HUI040009	China: Sichuan: Zhaojue	KX269210	/	KX269356
93	*Rana tagoi*	MSUZP-NPJP-R-08-69	Japan: Kyoto	KX269214	/	KX269359
94	*Rana taihangensis*	HNNU202008II001	China: Henan: Huixian	ON737935	ON738019	ON738116
95	*Rana temporaria*	ZMMU A-4288-1	Ukraine: Zakarpatska: Uzhgorod	KX269196	/	KX269343
96	*Rana tsushimensis*	NAP 4191	Japan: Nagasaki: Tsushima	KX269181	/	KX269329
97	*Rana ulma*	OKW 135	Japan: Ryukyu Islands	KX269215	/	KX269360
98	*Rana zhijinensis*	GZNU2018081606	China: Guizhou: Zhijin	OP577495	/	OP589319
99	*Rana zhenhaiensis*	KIZ 0803271	China: Zhejiang: Zhenhai	KX269218	JF939065	JF939105

For phylogenetic analysis, a total of 177 additional sequences were downloaded from GenBank (Table 2). *Rana shuchinae* Liu, 1950 was selected as outgroup based on Wan et al. (2020). Sequences were aligned using the algorithm MUSCLE with the default parameters (Edgar 2004), edited in order, and concatenated seriatim as a final dataset of 2515 bp (1042 bp for 16S, 558 bp for COI and 915 bp for cyt *b*), including 99 individuals across 44 species in MEGA 11 (Tamura et al. 2021). Phylogenetic analyses were conducted using Bayesian Inference (BI) and Maximum Likelihood (ML) methods in MrBayes v. 3.2.4 (Ronquist et al. 2012) and IQ-TREE v. 3.0.1 (Wong et al. 2025), respectively. For BI, two independent Markov chain Monte Carlo (MCMC) runs were performed for 200 million generations with sampling every 1,000 generations, and the first 25% of samples were discarded as burn-in; convergence was assessed with a potential scale reduction factor (PSRF) < 0.005 and ESS (effective sample size) value > 200 using Tracer 1.4 (http://tree.bio.ed.ac.uk/software/tracer/). For ML, the best-fit substitution model TPM2u+F+I+G4 was selected using ModelFinder based on the Bayesian Information Criterion (BIC) (Kalyaanamoorthy et al. 2017). A consensus tree was inferred from 1000 replicates each of the ultrafast bootstrap (UFB) and SH-aLRT (approximate likelihood ratio test) (Anisimova et al. 2011; Minh et al. 2013). Support values were considered strong when Bayesian posterior probabilities (BPP) ≥ 0.95, SH-aLRT ≥ 80, and UFB ≥ 95. Pairwise genetic distances were estimated in MEGA 11 (Tamura et al. 2021) based on three mitochondrial markers (16S, COI, and cyt *b*), employing the Kimura 2-parameter model with default settings. All phylogenetic trees were visualized and edited in TVBOT v2.6.1 (Xie et al. 2023).

## Results

### Morphological analyses

**Figure 2.**
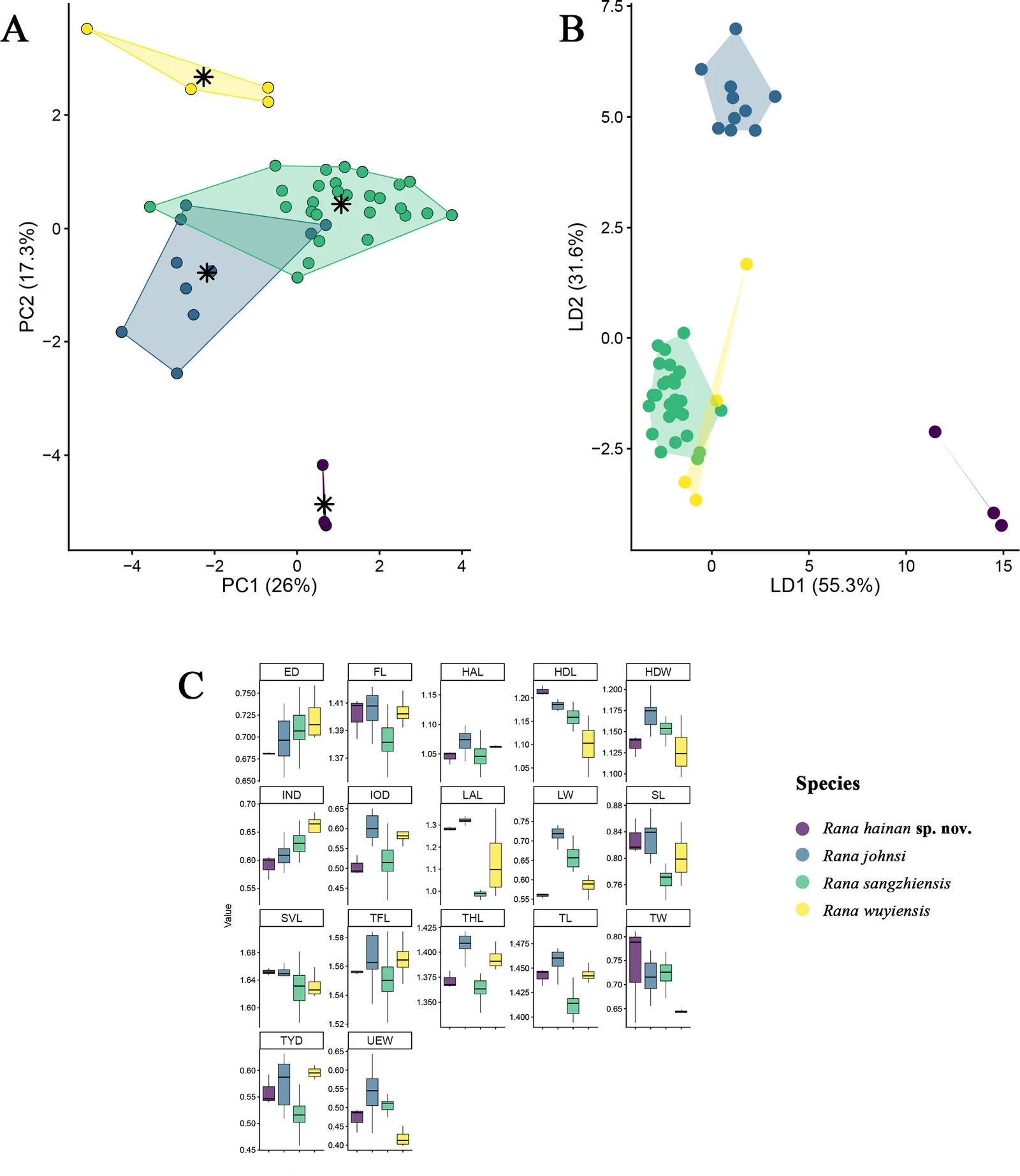
**A**. PCA; **B**. DAPC of morphometric data of male specimens in the *Rana johnsi* group; **C**. Box plots of morphometric data for each character in males.

Univariate comparisons of size-adjusted morphometric variables revealed significant differences among the four taxa, with the Hainan population clearly distinguishable from *Rana johnsi*, *R. sangzhiensis*, and *R. wuyiensis*. As shown in Table 3 and Fig. 2C, variables such as HDL, SL, IOD, UEW, ED, TYD, LW, THL, and TW exhibited significant pairwise differences between the Hainan population and the other taxa (p < 0.05, bold values), indicating clear morphological divergence.

**Table 3.** Results of the univariate comparisons of the size-corrected morphometric data. Significant differences (p < 0.05) are in bold. Abbreviations are in the Materials and methods.

**Species pairs**	**SVL**	**HDL**	**HDW**	**SL**	**IND**	**IOD**	**UEW**	**ED**	**TYD**	**LAL**	**HAL**	**LW**	**THL**	**TL**	**TW**	**TFL**	**FL**
*hainan* sp. nov. vs *johnsi*	1.0000	0.2816	0.5989	**0.0019**	0.1104	**0.0004**	**0.0146**	0.1139	0.5460	1.0000	1.0000	**0.0149**	**0.0046**	1.0000	**0.0063**	0.8639	0.9282
*hainan* sp. nov. vs *sangzhiensis*	0.5303	**0.0355**	0.7211	**0.0000**	1.0000	0.1552	0.0722	**0.0026**	0.9549	0.1311	0.3447	0.7144	0.3264	1.0000	0.1558	0.6186	0.6786
*hainan* sp. nov. vs *wuyiensis*	1.0000	**0.0006**	0.9278	**0.0002**	1.0000	**0.0015**	1.0000	**0.0050**	**0.0153**	1.0000	1.0000	0.0750	**0.0025**	1.0000	**0.0005**	0.1582	0.7865
*johnsi* vs *sangzhiensis*	0.1706	1.0000	0.9592	**0.0000**	0.0567	**0.0006**	1.0000	0.1516	**0.0129**	**0.0000**	0.9216	**0.0020**	**0.0291**	**0.0009**	0.1825	0.9249	0.8726
*johnsi* vs *wuyiensis*	1.0000	**0.0286**	0.1459	0.3984	**0.0102**	0.9947	**0.0036**	0.1884	0.0611	1.0000	1.0000	**0.0000**	1.0000	1.0000	0.8756	0.2457	0.2503
*sangzhiensis* vs *wuyiensis*	1.0000	0.0903	0.1820	**0.0456**	0.5667	**0.0126**	**0.0215**	0.8691	**0.0001**	**0.0496**	0.4246	**0.0001**	**0.0270**	**0.0125**	**0.0116**	0.3548	0.0593

Principal Component Analysis (PCA) of the size-adjusted and standardized dataset summarized the overall morphological variation among the four taxa. The first two principal components accounted for 43.3% of the total variance (PC1 = 26.0%, PC2 = 17.3%). In the PCA plot (Fig. 2A), the Hainan population was clearly separated from *R. johnsi*, *R. sangzhiensis*, and *R. wuyiensis*. Loadings indicate that TL, THL, and LAL contributed predominantly to PC1, whereas TW, HDL, and SL loaded heavily on PC2, reflecting the combined influence of overall body size and head/limb proportions on morphological differentiation (Table 4).

**Table 4.** Summary statistics and principal component analysis scores for the *Rana johnsi* group. Abbreviations are listed in the Materials and methods.

	**PC1**	**PC2**	**PC3**	**PC4**	**PC5**	**PC6**	**PC7**	**PC8**	**PC9**	**PC10**	**PC11**	**PC12**	**PC13**	**PC14**	**PC15**	**PC16**	**PC17**
SVL	-0.0872	-0.1904	-0.0453	-0.1789	0.0249	0.8603	-0.4026	0.0481	-0.0509	0.1181	0.0052	0.0012	-0.0054	0.0098	0.0058	0.0159	-0.0024
HDL	0.079	-0.4423	0.1313	0.0347	-0.2427	-0.0201	0.0447	-0.5237	-0.4099	-0.1177	0.1935	-0.2035	0.155	-0.0513	-0.2242	-0.2658	-0.189
HDW	-0.1332	-0.0877	0.5199	0.0907	0.2511	-0.0291	-0.0087	0.0179	0.3207	0.3587	0.2978	0.0832	-0.2935	-0.1307	-0.3582	-0.0431	-0.2669
SL	-0.2884	-0.3735	-0.1302	0.0828	0.2084	0.0111	0.2129	-0.077	0.1178	-0.1663	-0.0307	-0.1067	0.0787	0.4316	-0.1125	0.6088	-0.1708
IND	0.0322	0.0542	0.0436	0.4909	0.5368	0.1522	0.161	0.3059	-0.3312	-0.0281	0.0072	-0.3791	0.1398	-0.0159	0.0098	-0.2134	0.051
IOD	-0.3197	0.1951	-0.1117	-0.2511	-0.0862	0.0161	0.1932	0.2683	-0.4283	-0.1094	0.5889	0.1362	-0.0608	-0.1217	-0.2025	0.1737	0.1295
UEW	-0.0761	-0.0176	0.5585	-0.108	-0.0997	-0.031	-0.1527	0.1459	-0.1702	-0.4436	-0.3665	-0.2208	-0.2258	-0.2362	-0.0615	0.2739	0.1431
ED	-0.0066	0.3554	0.3055	0.206	0.2124	0.2066	0.017	-0.4794	0.0154	-0.3324	0.2175	0.3696	0.129	0.0484	0.307	0.1057	0.0314
TYD	-0.2822	0.0746	-0.22	-0.0926	0.4086	-0.1839	-0.2311	-0.3566	-0.3625	0.154	-0.3167	0.1801	-0.3705	0.0583	-0.1594	-0.0832	0.1249
LAL	-0.3405	-0.1987	-0.1094	-0.2232	0.1626	0.0543	0.1972	0.0382	0.3658	-0.5027	0.0289	-0.0149	-0.154	-0.0218	0.0639	-0.5416	0.0627
HAL	-0.2451	-0.2025	0.0305	0.1383	0.045	-0.3699	-0.7071	0.1616	0.0241	-0.0802	0.2949	-0.0109	0.2424	0.0791	0.2234	-0.0366	0.0595
LW	-0.1634	-0.2034	0.4294	-0.2599	-0.0026	-0.0395	0.2499	0.1974	-0.2516	0.2732	-0.1916	0.3091	0.2446	0.3647	0.2711	-0.1885	0.0698
THL	-0.3747	0.2522	0.095	-0.0309	-0.0863	0.0389	0.0245	-0.226	0.2327	0.1903	-0.0952	-0.2767	0.4746	-0.0241	-0.3322	-0.0293	0.4608
TL	-0.434	-0.0535	-0.0636	0.033	-0.0604	-0.0022	0.1697	-0.1448	-0.0287	0.2681	-0.0433	-0.2147	-0.0318	-0.5069	0.5535	0.1296	-0.2201
TW	0.0839	-0.4699	-0.0401	0.3606	-0.0702	0.0368	0.1382	-0.0183	0.0537	0.0861	0.0645	0.2846	-0.1773	-0.2199	0.0406	0.1025	0.6538
TFL	-0.2499	0.194	0.0627	0.3641	-0.4548	0.0922	-0.0037	-0.0193	-0.0303	0.0622	0.0729	-0.2155	-0.4618	0.4892	0.1227	-0.1427	0.0557
FL	-0.3091	0.0314	-0.0975	0.4288	-0.2551	0.0705	-0.0207	0.1845	-0.0493	-0.138	-0.3151	0.4628	0.2054	-0.1798	-0.2885	-0.0954	-0.3222
Eigenvalue	4.4176	2.9443	2.3536	1.7461	1.297	0.8967	0.7787	0.5616	0.4579	0.4091	0.3308	0.2556	0.2022	0.1465	0.0831	0.0616	0.0576
Proportion of Variance	0.2599	0.1732	0.1384	0.1027	0.0763	0.0528	0.0458	0.033	0.0269	0.0241	0.0195	0.015	0.0119	0.0086	0.0049	0.0036	0.0034
Cumulative Proportion	0.2599	0.433	0.5715	0.6742	0.7505	0.8033	0.8491	0.8821	0.909	0.9331	0.9526	0.9676	0.9795	0.9881	0.993	0.9966	1

Discriminant Analysis of Principal Components (DAPC) further confirmed clear morphological separation among the four taxa. Individuals of the Hainan population formed a completely isolated cluster with no overlap with other taxa, highlighting its distinct morphological identity. Convex hulls surrounding each group illustrate well-defined, species-specific clustering, supporting the recognition of the Hainan population as a morphologically independent entity (Fig. 2B).

Permutational multivariate analysis of variance (PERMANOVA) revealed significant morphological differentiation for most pairwise comparisons (Table 5). The Hainan population differed significantly from *R. johnsi* (F = 7.971, R^2^ = 0.420, p_adjusted = 0.0306) and *R. sangzhiensis* (F = 11.336, R^2^ = 0.288, p_adjusted = 0.0006), but not from *R. wuyiensis* (F = 11.786, R^2^ = 0.702, p_adjusted = 0.1714). All other pairwise comparisons were significant following Bonferroni correction.

**Table 5.** Results of the PERANOVA of the PCA. Significant differences are in bold.

**Species pairs**	**F_Model**	**R2**	**p_value**	**p_adjusted**
*hainan* sp. nov. vs *johnsi*	7.9710	0.4202	0.0051	**0.0306**
*hainan* sp. nov. vs *sangzhiensis*	11.3366	0.2882	0.0001	**0.0006**
*hainan* sp. nov. vs *wuyiensis*	11.7868	0.7021	0.0286	0.1714
*johnsi* vs *sangzhiensis*	10.7490	0.2350	0.0001	**0.0006**
*johnsi* vs *wuyiensis*	6.2730	0.3433	0.0010	**0.0060**
*sangzhiensis* vs *wuyiensis*	10.3138	0.2623	0.0001	**0.0006**

Collectively, these results indicate that the Hainan population is morphologically distinct from most of its congeners.

### Phylogenetic analyses

**Figure 3.**
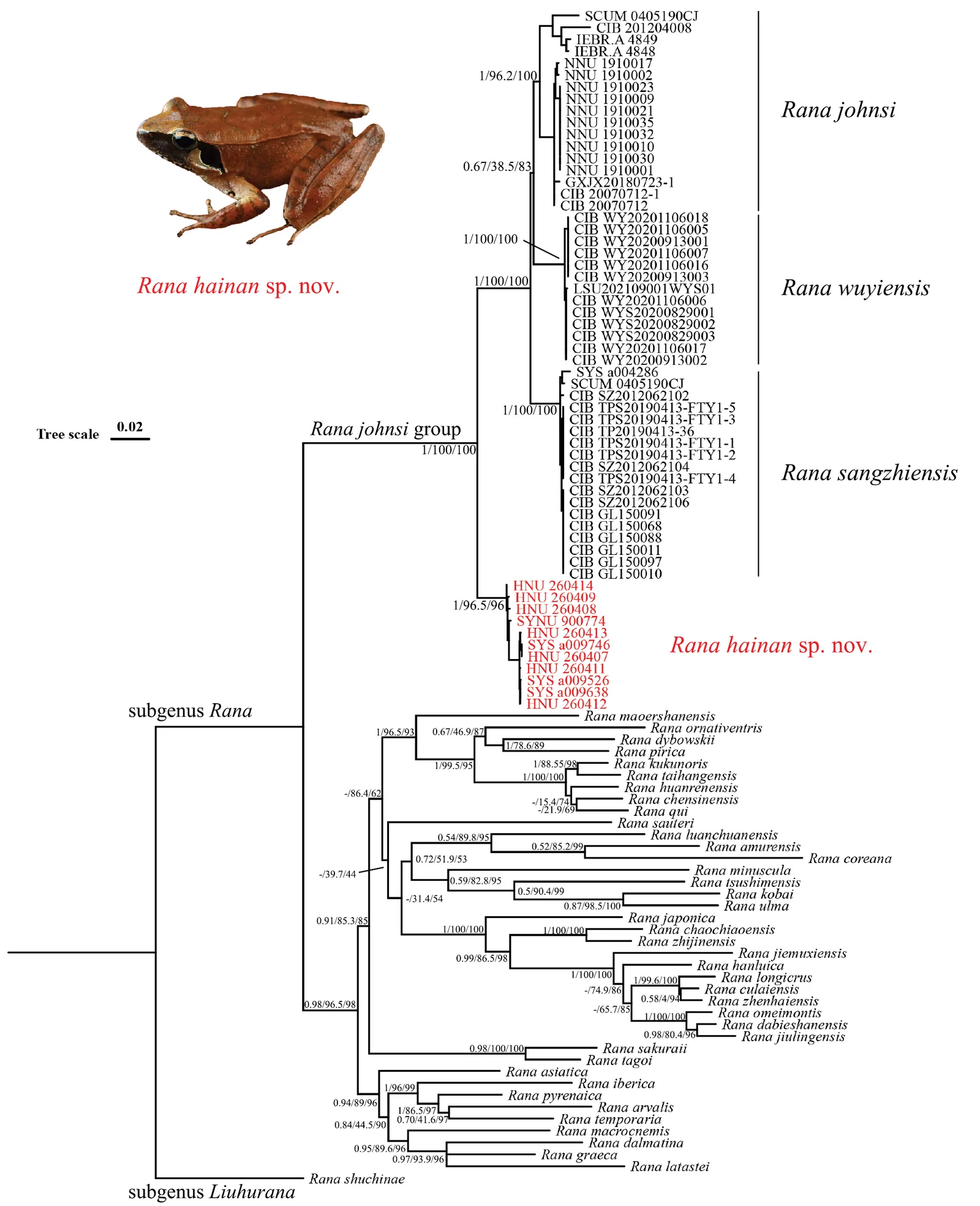
Maximum Likelihood consensus tree inferred from 16S, COI, and cyt *b* genes. Node labels indicate BPP/SH-aLRT/UFB.

The BI and ML analyses produced identical topologies, which are summarized in Fig. 3. Pairwise genetic distances based on 16S, COI and cyt *b* among all species are provided in the Suppl. material 1–3, respectively.

Phylogenetic analyses recovered a strongly supported monophyletic clade comprising all samples of the *Rana johnsi* group (BPP = 1.00; SH-aLRT = 100; UFB = 100). Within this clade, well-supported lineages corresponding to *R. johnsi*, *R. wuyiensis*, *R. sangzhiensis*, and the Hainan population were resolved. Importantly, the Hainan population forms a distinct, strongly supported lineage (BPP = 1.00; SH-aLRT = 96.5; UFB = 96) that is sister to the clade containing *R. johnsi*, *R. wuyiensis*, and *R. sangzhiensis*. The Hainan lineage exhibits pronounced genetic divergence from all recognized congeners used in this study (Kimura 2-parameter distances: 16S 1.50–10.85%, COI 5.90–20.09%, cyt *b* 7.37–21.29%), consistent with its distinct evolutionary lineage.

Based on its phylogenetic position, high genetic divergence, and morphological distinctiveness (see Morphological analyses above), we consider them to be all separate species and describe the specimens from Wanning and Mt. Diaoluo, Hainan as a new species.

### Taxonomic account

#### Rana hainan

Taxon classificationAnimaliaAnuraRanidae

Wang, Qiu, Song, Qi, Wang, Li & Wang sp. nov.

8359A660-02F1-540A-9E44-F7A984781D79

https://zoobank.org/7C07DF97-D1F2-4FE3-B043-E67793DAE960

Table 6; Figs 4, 5

Chresonymy. Rana nigrotympanica – Wang et al. (2007).
Rana johnsi – Shi et al. (2011, in part).

##### Type material.

***Holotype***: China • 1 ♂; Hainan Province, Wanning City, Sangengluo Town, Shangxi Provincial Nature Reserve, 18.795246°N, 110.162373°E; altitude 350 m a.s.l.; collected by MHH on 16 January 2026; HNU 260411. ***Paratypes* (n=11)**: China • 2 ♂; same locality as holotype; collected by MHH on 09 October 2025 and 12 April 2026; SYS a009638, HNU 260412 • 5 ♀; same locality as holotype; collected by MHH on 04 September 2025 and 12 April 2026, and by MHH and PPL on 11 January 2024; HNU 250901, SYS a009526, SYS a009527 (Field NO. SYNU 900774), HNU 260413–14• 4 ♀; Hainan Province, Lingshui County, Mt. Diaoluo, 18.7248115°N, 109.8770934°E; altitude 950 m a.s.l.; collected by CXL and DED on 19 April 2026; SYS a009746, HNU 260407–09.

##### Diagnosis.

*Rana hainan* sp. nov. is distinguished by the following morphological characteristics: (1) body medium-sized, SVL 44.4–45.4 (44.9 ± 0.5, *n* = 3) mm in adult males, 45.4–57.5 (51.2 ± 4.2, *n* = 9) mm in adult females; (2) head length significantly larger than head width; (3) a distinct triangular black patch on the temporal region; (4) dorsolateral fold distinct and thin, extending straight from posterior margin of upper eyelid to above groin; (5) internarial distances larger than interorbital distances; (6) eyes relatively small; (7) tympanum diameter significantly smaller than eye diameter; (8) fingers without lateroventral grooves, unwebbed, relative finger lengths II < I < IV < III; (9) presence of supernumerary tubercles below the bases of each finger, presence of three separated metacarpal tubercles; (10) toe tips slightly expanded into small discs, lateroventral grooves present on all toe tips, connected at the anterior margin, toes webbing formula: I 0–1/2 II 0–1/2 III 1^-^ – 1/2 IV 1^-^ – 0 V, relative toe lengths I < II < III < V < IV; (11) tibio-tarsal articulation reaching forward beyond tip of snout; (12) heels overlapping; (13) dorsal skin smooth, flanks smooth with few granules; (14) presence of vocal sacs in males; (15) breeding males possessing a grayish-white nuptial pad forming a continuous patch on finger I, extending from the tip of the finger to the posterior margin of the inner metacarpal tubercle, and densely covered with tiny hoary nuptial spines.

##### Description of the holotype

**Figure 4.**
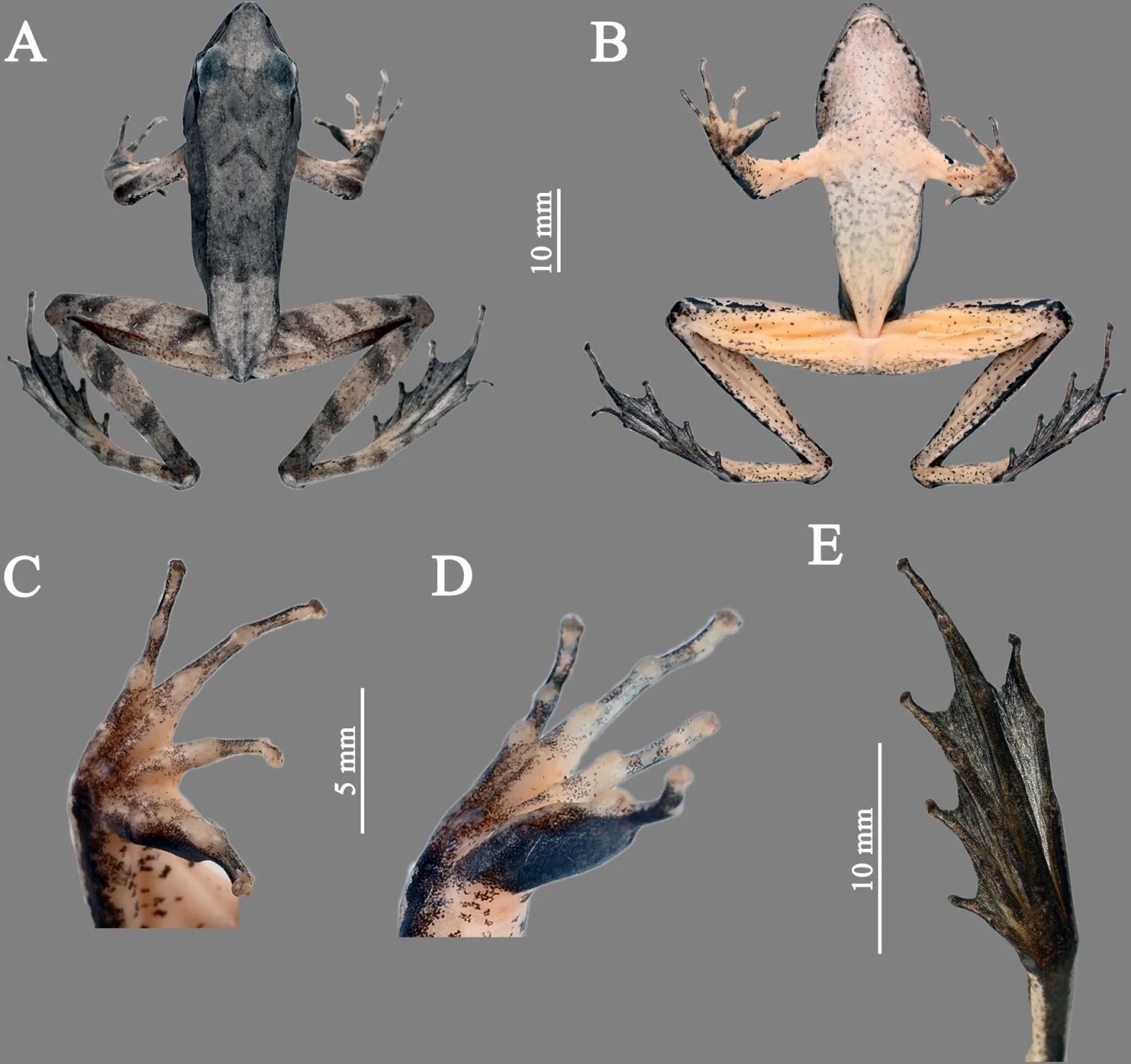
Morphological features of the adult male holotype HNU 260411 of *Rana hainan* sp. nov. **A**. Dorsal view of body; **B**. Ventral view of body; **C**. Ventral surface of right hand; **D**. Showing the nuptial pad and nuptial spines; **E**. Ventral surface of left foot. Photos by HTW.

**(Fig. 4)**. Adult male. Moderate body size, SVL 44.8 mm; head length larger than head width, HDW/HDL 0.91; snout slightly pointed in dorsal view, projecting, sloping backward to mouth in profile, protruding well beyond margin of lower jaw; top of head flat; eyes moderate in size, ED/HDL 0.27, pupil oval and horizontal; nostril oblique-ovoid; canthus rostralis well developed; loreal region slightly oblique; internasal distance larger than interorbital distance, IND/IOD 1.09; tympanum boundary clear, tympanic rim feebly elevated, ED/TYD 1.23; a skin fold present posterior to tympanum, disconnected with dorsolateral fold, swollen near shoulder; large ovoid choanae at the base of the maxilla; vomerine teeth in two short rows, oblique and separated by a distance about one row of teeth; maxillary teeth present; tongue deeply notched posteriorly; a pair of internal subgular vocal sacs present, openings slit like, small, positioned at on inner mandible near the corners of mouth.

Forearms 0.19 of SVL and hand 0.24 of SVL; fingers slender, without web but with narrow fringe; tip of fingers rounded, not expanded, without lateroventral grooves; relative finger lengths II < I < IV < III; subarticular tubercles significantly prominent, rounded, formula 1, 1, 2, 2; distinct, small, rounded supernumerary tubercles below the bases of each finger; inner metacarpal tubercle indistinct, ovoid, partly covered by nuptial pad; two outer metacarpal tubercles distinctly separated, slightly larger, long elliptic; nuptial pad with tiny spines on the finger I, extending from the tip of the finger to the posterior margin of the inner metacarpal tubercle.

Hindlimbs long, tibia 0.60 of SVL and length of foot and tarsus 0.79 ratio of SVL; thigh shorter than tibia, heels overlap when hindlimbs flexed at right angles to axis of body; tibio-tarsal articulation reaching far beyond snout when hindlimb stretched forward along body; toe tips slightly expanded into small discs with lateroventral grooves present and connected at middle of front edge; inner edge of toe I and outer edge of toe V with narrow lateral fringe, relative toe lengths I < II < III < V < IV, toes webbing formula: I 0–1/2 II 0–1/2 III 1^-^ – 1/2 IV 1^-^ – 0 V; subarticular tubercles prominent and oval, formula 1, 1, 2, 3, 2; supernumerary tubercles absent; inner metatarsal tubercle oval and prominent, outer metatarsal tubercle rounded, indistinct.

Dorsal skin smooth, supratympanic fold absent; dorsum with a distinct chevron-shaped skin ridge; dorsolateral folds distinct, narrow, straight, extending from edge of upper eyelid to hip, not curve above tympanum; several small tubercles on flank; several tiny granules on the skin of loreal and temporal regions; ventral skin smooth, skins around cloaca with numerous flat tubercles; skin on limbs with transverse parallel bars; tarsal fold absent, and small tubercles present on the tarsus.

##### Coloration in life.

In life, dorsal surface overall reddish brown; a transverse stripe formed by black speckles present between the upper eyelids; loreal region dark brown, with white spots along the lower margin; a distinct triangular black patch present on temporal region, completely covering the tympanum, with its anterior margin edged by a pale border; iris bicolored, upper one-third pale yellowish brown and lower part darker, separated by a distinct horizontal boundary; pupil dark brown. Dorsal surfaces of limbs reddish brown, with several dark transverse bars; ventral surface of forelimb brown near the junction with posterior dorsal surface, gradually becoming paler toward anterior side; fingers grey; nuptial pad greyish white; toe webs dark brown to black; toe tips grey; ventral surface of tibia yellow; posterior surface of thigh orange brown. Ventral surface creamy white to pale yellow; throat and chest suffused with pale yellow.

In preservative, dorsal surface greyish brown with dark brown mottling; limbs greyish brown with dark transverse bars; loreal and temporal regions dark grey; ventral surface creamy white, with grey mottling on throat and chest; ventral surfaces of limbs pale orange to beige, scattered with dark speckles; toe webs dark grey to black; nuptial pad dark grey.

##### Variation.

**Figure 5.**
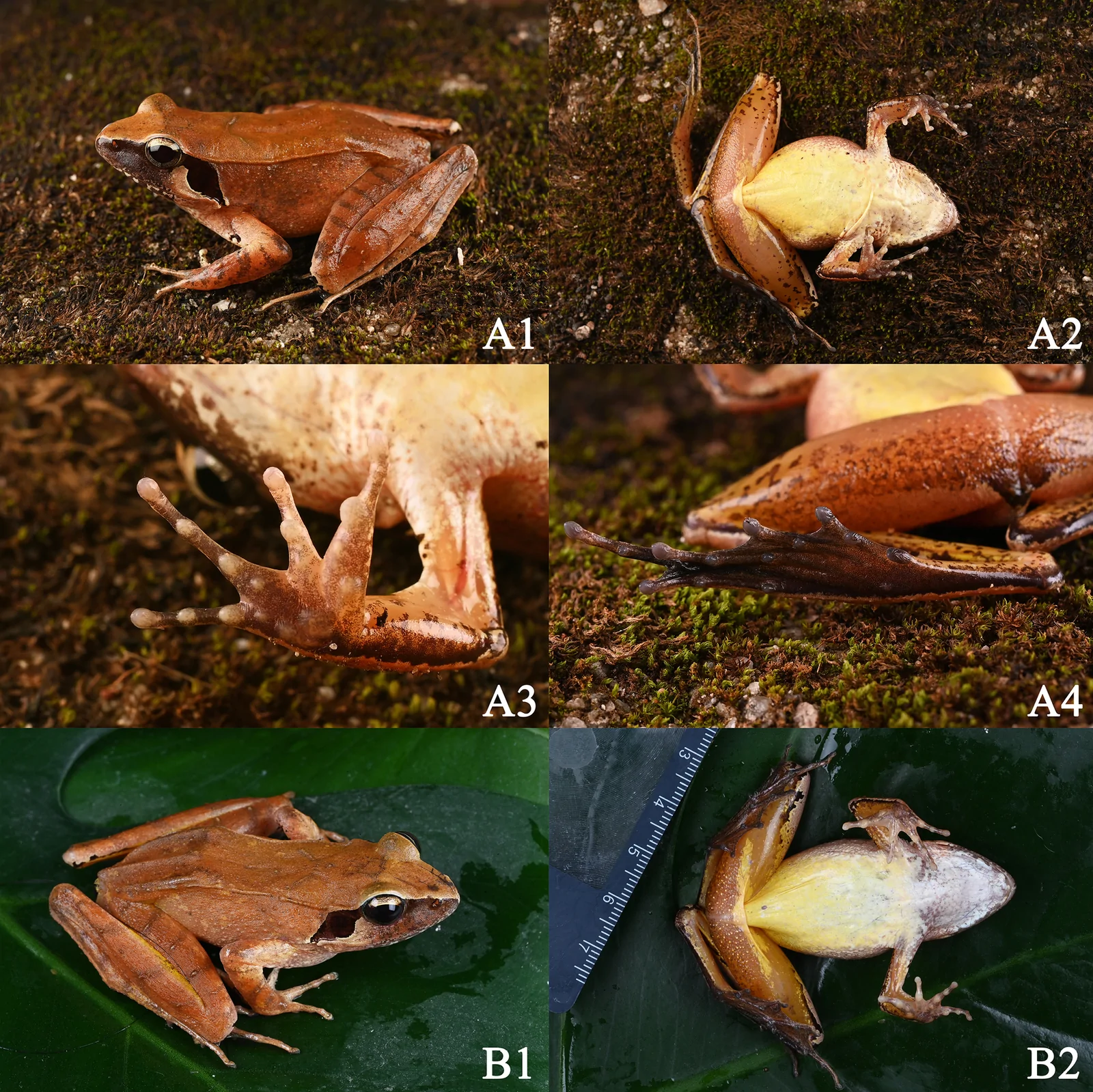
The female paratypes of *Rana hainan* sp. nov. in life. **A**. SYS a009526; **B**. SYS a009527 (Field NO. SYNU 900774); 1. Dorsolateral view; 2. Ventral view; 3. Palmar surface of right hand; 4. Plantar surface of right foot. Photos by HTW and XCQ.

Measurements of type series specimens are given in Table 6. Dorsolateral folds are discontinuous in the female paratype SYS a009527 (Field NO. SYNU 900774) (Fig. 5B). The number of transverse bars ranges from 2–4 on forearms, 3–5 on thigh, and 3–6 on tibia.

**Table 6.** Measurements (in mm) and body proportions of the type series of *Rana hainan* sp. nov. The asterisk indicates the holotype.

**Voucher ID**	**HNU 251008**	HNU 260411*	**HNU 260412**	**SYNU 900774**	**HNU 250901**	**SYS a009526**	**HNU 260413**	**HNU 260414**	**SYS a009746**	**HNU 260407**	**HNU 260408**	**HNU 260409**
**Sex**	**Male**	**Male**	**Male**	**Female**	**Female**	**Female**	**Female**	**Female**	**Female**	**Female**	**Female**	**Female**
SVL	44.4	44.8	45.4	51.4	54.3	56.0	50.8	48.5	57.5	46.2	45.4	50.4
HDL	16.4	16.9	15.9	19.0	19.5	19.9	19.0	17.9	20.6	18.8	17.7	18.7
HDW	14.0	13.2	13.7	17.2	16.7	18.5	15.5	15.4	17.9	15.7	14.4	16.6
SL	6.8	7.3	6.2	7.4	7.8	8.9	7.4	6.7	8.6	7.4	7.0	7.3
IND	4.2	3.7	3.8	4.2	4.8	4.9	4.4	3.9	4.8	4.5	4.2	4.6
IOD	3.0	3.4	3.2	3.3	4.0	4.1	3.8	3.4	4.3	3.9	3.5	3.6
UEW	3.0	2.7	3.2	3.2	3.5	4.3	3.0	3.3	4.3	3.3	3.0	3.4
ED	4.7	4.8	4.9	5.1	5.3	5.6	5.3	5.0	6.2	4.7	4.7	5.3
TYD	3.4	3.9	3.6	3.9	3.7	4.4	3.7	3.5	3.8	3.5	3.2	4.0
LAL	18.1	19.5	20.1	21.5	20.5	23.2	23.8	21.8	26.1	20.5	21.8	22.8
HAL	11.5	10.8	11.0	13.0	12.1	14.2	11.9	11.6	14.2	11.8	12.6	12.4
LW	3.9	3.6	3.4	3.1	4.0	4.0	3.1	3.0	3.9	3.2	3.4	3.5
THL	22.7	24.0	23.9	28.3	29.6	32.5	28.1	25.1	30.2	26.5	25.6	27.9
TL	27.8	27.0	28.2	32.9	32.1	35.4	32.8	31.0	33.9	30.7	31.1	32.4
TW	7.9	4.3	4.9	4.1	5.4	5.7	5.7	4.2	6.1	4.2	4.4	5.4
TFL	35.7	35.8	36.4	40.7	38.5	45.5	43.2	37.7	43.0	37.9	38.2	41.4
FL	25.7	24.2	25.7	28.6	26.6	30.7	30.0	24.6	29.4	25.4	26.1	29.0
HDL/SVL	0.37	0.38	0.35	0.37	0.36	0.36	0.37	0.37	0.36	0.41	0.39	0.37
HDW/HDL	0.85	0.78	0.86	0.91	0.86	0.93	0.82	0.86	0.87	0.84	0.81	0.89
SL/SVL	0.15	0.16	0.14	0.14	0.14	0.16	0.15	0.14	0.15	0.16	0.15	0.14
ED/HDL	0.29	0.28	0.31	0.27	0.27	0.28	0.28	0.28	0.30	0.25	0.27	0.28
TYD/HDL	0.21	0.23	0.23	0.21	0.19	0.22	0.19	0.20	0.18	0.19	0.18	0.21
TL/SVL	0.63	0.60	0.62	0.64	0.59	0.63	0.65	0.64	0.59	0.66	0.69	0.64
THL/SVL	0.51	0.54	0.53	0.55	0.55	0.58	0.55	0.52	0.53	0.57	0.56	0.55
TFL/SVL	0.80	0.80	0.80	0.79	0.71	0.81	0.85	0.78	0.75	0.82	0.84	0.82
FL/SVL	0.58	0.54	0.57	0.56	0.49	0.55	0.59	0.51	0.51	0.55	0.57	0.58
HAL/SVL	0.26	0.24	0.24	0.25	0.22	0.25	0.23	0.24	0.25	0.26	0.28	0.25
LAL/SVL	0.41	0.44	0.44	0.42	0.38	0.41	0.47	0.45	0.45	0.44	0.48	0.45

##### Sexual dimorphism.

Breeding males possessing a grayish-white nuptial pad forming a continuous patch on finger I, extending from the tip of the finger to the posterior margin of the inner metacarpal tubercle, and densely covered with tiny hoary nuptial spines. Male with a pair of internal subgular vocal sacs.

##### Etymology.

The specific epithet “*hainan*” is a noun in apposition referring to the type locality of Hainan Province, China. The English common name “Hainan Groove-toed Frog” and formal Chinese name “海南趾沟蛙” (hǎi nán zhǐ gōu wā) are proposed.

##### Comparison.

*Rana hainan* sp. nov. is assigned to the *R. johnsi* group due to the presence of lateroventral grooves on the flattened tips of the toes, a distinct triangular black patch on the temporal region, and vocal sacs in males, distinguishing it from other species of the subgenus *Rana*.

Within the *R. johnsi* group, *Rana hainan* sp. nov. differs from *R. johnsi* in mean values of SL, IOD, UEW, LW, THL, TW (Table 3), and by the smaller HDW/HDL in males (0.78–0.86 vs 0.93–1.03; Suppl. material 4); differs from *R. sangzhiensis* in mean values of HDL, SL, ED (Table 3), by the smaller HDW/HDL in males (0.78–0.86 vs 0.91–1.08; Suppl. material 4), by the toes webbing formula: I 0–1/2 II 0–1/2 III 1- – 1/2 IV 1- – 0 V (vs entirely webbing), and by the tibio-tarsal articulation reaching far beyond snout when hindlimb stretched forward along body (vs reaching the nostril or tip of the snout); differs from *R. wuyiensis* in mean values of HDL, SL, IOD, ED, TYD, THL, TL (Table 3), by the smaller HDW/HDL, ED/HDL and TYD/HDL in females (0.81–0.93 vs 0.97–1.26, 0.25–0.30 vs 0.36–0.44, and 0.18–0.22 vs 0.25–0.36, respectively; Suppl. material 4), by the tarsal fold absent (vs presence), by the connect lateroventral grooves on toes (vs unconnected), by the nuptial pad forming a continuous patch on finger I in males (vs divided into three parts), and by the toes webbing formula: I 0–1/2 II 0–1/2 III 1^-^ – 1/2 IV 1^-^ – 0 V (vs 1 ^1^/_3_–2 II 1 ^1^/_3_–2 ^1^/_3_ III 1 ½ – 2 ^2^/_3_ IV 3–1 ^1^/_3_ V).

##### Distribution and natural history.

*Rana hainan* sp. nov. is currently known from Shangxi Provincial Nature Reserve in Wanning and the Diaoluo Mountain section of Hainan Tropical Rainforest National Park, Hainan, China. The population reported from Mt. Jianfengling, Ledong by Fei et al. (2009) was tentatively identified as *Rana cf. johnsi* prior to the collection of specimens (see Discussion below).

**Figure 6.**
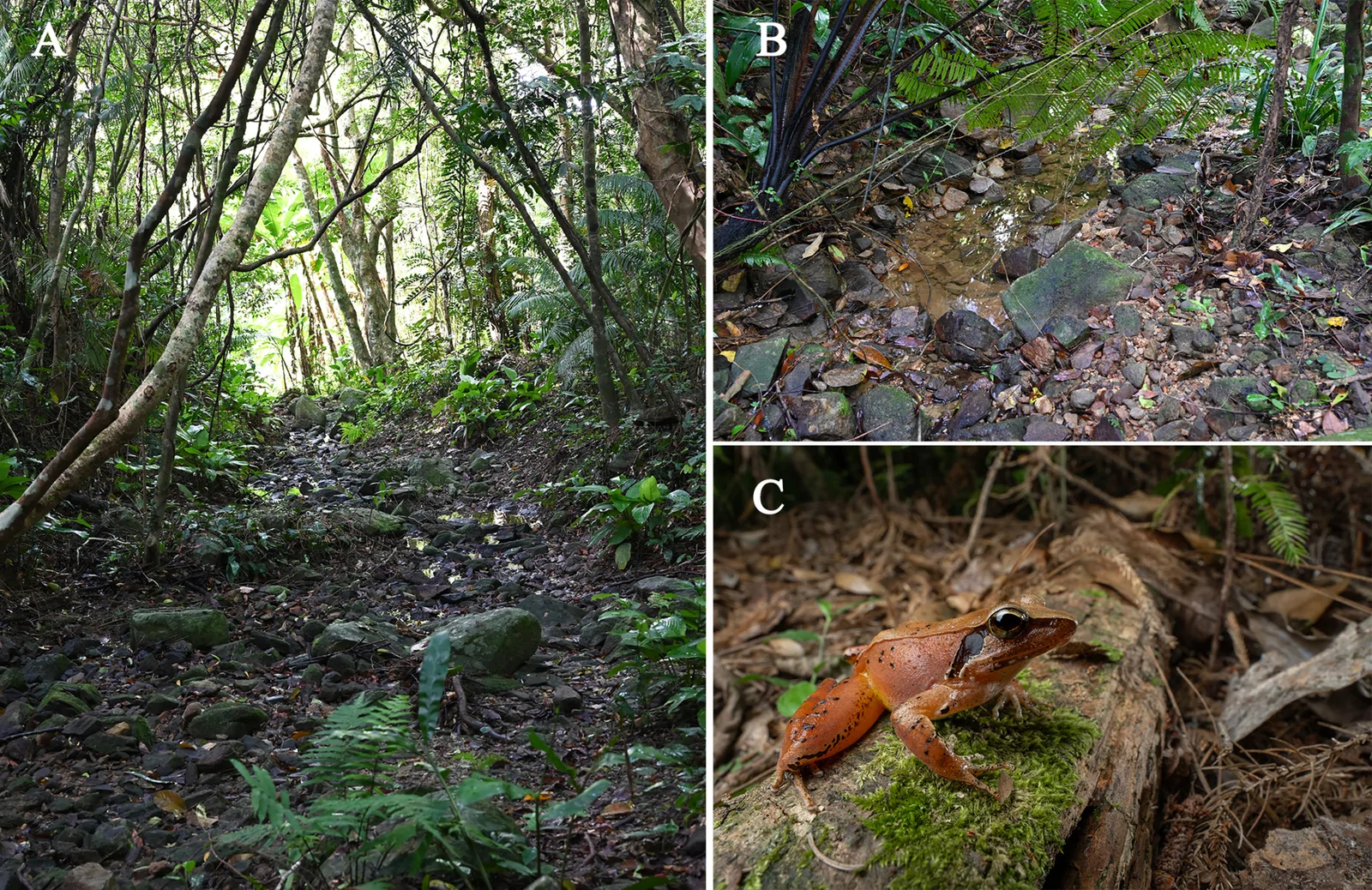
**A**. Habitat of *Rana hainan* sp. nov. in Shangxi Provincial Nature Reserve; **B**. Microhabitat of *Rana hainan* sp. nov. in Shangxi Provincial Nature Reserve; **C**. Female Paratype SYS a009746 *Rana hainan* sp. nov. from Mt. Diaoluo in its natural habitat. Photos by HTW and CXL.

*Rana hainan* sp. nov. inhabits tropical rainforest at elevations of 350–950 m, predominantly in evergreen broadleaf forests (Fig. 6). The understory is rich in ferns and shrubs, and streams are typically rocky. Breeding often occurs in seasonal pools and slow-flowing backwaters of these streams. Tadpoles were observed in November at microhabitats similar to that shown in Fig. 6B (Li, pers. obs.). Male specimens collected from October to April consistently exhibited nuptial pads and nuptial spines, suggesting that the breeding season likely occurs during this period. Furthermore, dissection of a female specimen (SYS a009746) collected in April revealed an empty abdominal cavity and contracted ovaries, indicating that this time corresponds to the late stage of the reproductive season.

## Discussion

The phylogenetic relationships recovered within the *Rana johnsi* group reveal a clear geographic structure across southern China and Vietnam. In the reconstructed phylogeny, *Rana hainan* sp. nov. is clearly separated from the remaining congeners, followed by *R. sangzhiensis*, whereas *R. johnsi* and *R. wuyiensis* form a strongly supported sister clade (Fig. 3). Geographically, *R. johnsi* is distributed from central Vietnam to Guangxi, southern China, *R. sangzhiensis* occurs mainly in central and southwestern China including Hunan and Sichuan, and *R. wuyiensis* is restricted to the Wuyi Mountains of Fujian, China (Wu et al. 2021). Different lineages of the group are distributed in geographically separated montane regions, collectively exhibiting a clear disjunct distribution pattern. This pattern suggests that geographic isolation may have played an important role in the diversification of the group. The distinct phylogenetic position of *Rana hainan* sp. nov., together with its diagnostic morphological characters, supports its recognition as a distinct species endemic to Hainan Island.

In light of the taxonomic confusion surrounding historical *Rana* records from Hainan Island outlined above, our results provide new evidence for reassessing the identity of these populations. Most previous studies lack clear voucher specimens from Hainan or detailed identification information, and *R. johnsi* and *R. nigrotympanica* are morphologically similar, making misidentifications likely in the absence of systematic comparisons. Furthermore, the taxonomic status of *R. nigrotympanica* has long been controversial, with Fei et al. (2009, 2012) recognizing it as a distinct species, whereas Ohler (2007) treated it as a junior subjective synonym of *Hylarana cubitalis* (Smith, 1917). *Rana nigrotympanica* was established by Dubois (1992), based on the specimen figured in Liu and Hu (1959: fig. 4), which was subsequently designated as CIB 571162 by Ohler (2007). The type locality was not specified beyond “Chine” (French for “China”) in the original treatment, but Ohler (2007) provided “Mengyan (21°37'N, 101°22'E), Xishuangbanna Dai Autonomous Prefecture, Yunnan Province, China” as the type locality in her synonymy. Following Ohler (2007), *R. nigrotympanica* is currently considered a junior synonym of *Hylarana cubitalis* (Frost 2026; AmphibiaChina 2026), with the latter not known from Hainan (AmphibiaChina 2026). Therefore, previous records of *R. nigrotympanica* from Hainan require re-evaluation. *Rana johnsi* was originally described as Rana sauteri var. johnsi by Smith (1921), based on six syntypes from Sui Kat, Langbian Plateau, Vietnam. The taxon was subsequently treated as a subspecies of *Hylarana sauteri* (Bourret 1939), and as *Pseudorana johnsi* (Jiang et al. 1997), before being treated as *Rana johnsi* by Inger et al. (1999) and Frost et al. (2006). It is currently placed in the subgenus *Rana* (Yuan et al. 2016). Although *R. hainan* sp. nov. belongs to the *R. johnsi* group, it can be distinguished from *R. johnsi* by several diagnostic morphological characters (see Diagnosis), and the two taxa are also recovered as distinct evolutionary lineages in our phylogenetic analyses. Based on the combined morphological and molecular evidence, the population from Mt. Diaoluo is herein recognized as representing an undescribed species, *Rana hainan* sp. nov., and previous records from Mt. Diaoluo are accordingly referred to this new taxon. For the remaining historical records from Hainan, however, the available evidence is insufficient to assign them confidently to either *R. hainan* sp. nov. or *R. johnsi*. Given that populations from different regions of Hainan may exhibit significant genetic differentiation (Lin et al. 2010; Huang et al. 2013, 2019; Qi et al. 2025), and that other localities currently lack verifiable voucher specimens or molecular data, these remaining historical records are conservatively treated as *Rana cf. johnsi* pending further study.

## Supplementary Material

XML Treatment for Rana hainan

## Additional information

### Artificial Intelligence (AI) use

The authors accept full responsibility for the content of the manuscript, including the disclosure of any use of AI.

No AI tools were used in the preparation of this manuscript.

### Author ORCIDs

H.-T. Wang https://orcid.org/0000-0002-2415-3234

M.-H. Huang https://orcid.org/0009-0002-9965-9309

H.-M. Song https://orcid.org/0009-0005-8244-1567

S. Qi https://orcid.org/0000-0002-2924-6093

W. Wang https://orcid.org/0009-0002-6437-0484

B. Wang https://orcid.org/0000-0001-6036-5579

Y.-Y. Wang https://orcid.org/0000-0003-2024-6641

P.-P. Li https://orcid.org/0000-0002-0713-5384

J.-C. Wang https://orcid.org/0000-0001-5081-2355

### Data availability

All of the data that support the findings of this study are available in the main text or Supplementary Information.

## Supplementary materials

Supplementary material 1 Kimura 2-parameter distances (%) of the 16S gene amongst species of *Rana* used in this study Data type xlsx https://binary.pensoft.net/file/1828049This dataset is made available under the Open Database License (http://opendatacommons.org/licenses/odbl/1.0/). The Open Database License (ODbL) is a license agreement intended to allow users to freely share, modify, and use this Dataset while maintaining this same freedom for others, provided that the original source and author(s) are credited. Hao-Tian Wang, Ming-Hong Huang, Chen-Xi Liao, Xian-Chun Qiu, Han-Ming Song, Shuo Qi, Da-Er Ding, Shang-Biao Zhuo, Wei Wang, Jun Wu, Bin Wang, Ying-Yong Wang, Pi-Peng Li, Ji-Chao Wang

Supplementary material 2 Kimura 2-parameter distances (%) of the COI gene amongst species of *Rana* used in this study Data type xlsx https://binary.pensoft.net/file/1828050This dataset is made available under the Open Database License (http://opendatacommons.org/licenses/odbl/1.0/). The Open Database License (ODbL) is a license agreement intended to allow users to freely share, modify, and use this Dataset while maintaining this same freedom for others, provided that the original source and author(s) are credited. Hao-Tian Wang, Ming-Hong Huang, Chen-Xi Liao, Xian-Chun Qiu, Han-Ming Song, Shuo Qi, Da-Er Ding, Shang-Biao Zhuo, Wei Wang, Jun Wu, Bin Wang, Ying-Yong Wang, Pi-Peng Li, Ji-Chao Wang

Supplementary material 3 Kimura 2-parameter distances (%) of the cyt *b* gene amongst species of *Rana* used in this study Data type xlsx https://binary.pensoft.net/file/1828051This dataset is made available under the Open Database License (http://opendatacommons.org/licenses/odbl/1.0/). The Open Database License (ODbL) is a license agreement intended to allow users to freely share, modify, and use this Dataset while maintaining this same freedom for others, provided that the original source and author(s) are credited. Hao-Tian Wang, Ming-Hong Huang, Chen-Xi Liao, Xian-Chun Qiu, Han-Ming Song, Shuo Qi, Da-Er Ding, Shang-Biao Zhuo, Wei Wang, Jun Wu, Bin Wang, Ying-Yong Wang, Pi-Peng Li, Ji-Chao Wang

Supplementary material 4 Morphometric comparisons between the *Rana hainan* sp. nov. and its closely related species Data type docx https://binary.pensoft.net/file/1828052This dataset is made available under the Open Database License (http://opendatacommons.org/licenses/odbl/1.0/). The Open Database License (ODbL) is a license agreement intended to allow users to freely share, modify, and use this Dataset while maintaining this same freedom for others, provided that the original source and author(s) are credited. Hao-Tian Wang, Ming-Hong Huang, Chen-Xi Liao, Xian-Chun Qiu, Han-Ming Song, Shuo Qi, Da-Er Ding, Shang-Biao Zhuo, Wei Wang, Jun Wu, Bin Wang, Ying-Yong Wang, Pi-Peng Li, Ji-Chao Wang
