# Supplementary material for: A new species of Rana (Amphibia, Anura, Ranidae) from Hainan, Southern China
Source: Zookeys. 2026 Nov 4;1296:185–208. doi: 10.3897/zookeys.1296.211503 (PMC13755813; doi:10.3897/zookeys.1296.211503)
Supplement: Supplementary file 1 [file zookeys-1296-185_article-211503__-s004.docx]

**Table S4.** Morphometric comparisons between the *Rana hainanensis* **sp. nov.** and its closely related species. Units given in mm. Abbreviations are in the Materials and Method.

| Species | | *Rana hainanensis* sp. nov. | | *R. johnsi* | | *R. sangzhiensis* | *R. wuyiensis* | |
| --- | --- | --- | --- | --- | --- | --- | --- | --- |
| Sex | | Male (n = 3) | Female (n = 9) | Male (n = 10) | Female (n = 1) | Male (n = 27) | Male (n = 4) | Female (n = 2) |
| SVL | Range | 44.4–45.4 | 45.4–57.5 | 40.7–47.2 | 54.4 | 37.9–51.4 | 41.4–45.6 | 47.6–50.3 |
|  | Mean ± SD | 44.9 ± 0.5 | 51.2 ± 4.2 | 44.62± 1.7 | / | 43.0 ± 2.9 | 42.9 ± 1.9 | 49.0 ± 1.9 |
| HDL | Range | 15.9–16.9 | 17.7–20.6 | 14.0–16.2 | 18.1 | 12.4–16.6 | 10.3–14.7 | 12.8–15.5 |
|  | Mean ± SD | 16.4 ± 0.5 | 19.0 ± 0.9 | 15.1 ± 0.8 | / | 14.4 ± 0.9 | 12.8 ± 2.2 | 14.2 ± 1.9 |
| HDW | Range | 13.2–14.0 | 14.4–18.5 | 13.3–15.9 | 17.0 | 13.1–15.9 | 12.3–15.0 | 15.0–16.1 |
|  | Mean ± SD | 13.6 ± 0.4 | 16.4 ± 1.3 | 14.8 ± 0.9 | / | 14.2 ± 0.7 | 13.5 ± 1.3 | 15.6 ± 0.8 |
| SL | Range | 6.2–7.3 | 6.7–8.9 | 6.2–7.5 | 7.3 | 5.2–7.2 | 5.7–7.1 | 6.7–7.4 |
|  | Mean ± SD | 6.8 ± 0.6 | 7.6 ± 0.7 | 6.8 ± 0.5 | / | 5.9 ± 0.4 | 6.4 ± 0.7 | 7.1 ± 0.5 |
| IND | Range | 3.7–4.2 | 3.9–4.9 | 3.5–4.6 | 4.3 | 3.4–5.1 | 4.1–5.0 | 4.9–7.7 |
|  | Mean ± SD | 3.9 ± 0.3 | 4.5 ± 0.3 | 4.1 ± 0.3 | / | 4.3 ± 0.4 | 4.6 ± 0.4 | 6.3 ± 2.0 |
| IOD | Range | 3.0–3.4 | 3.3–4.3 | 3.5–4.9 | 5.4 | 2.6–4.2 | 3.5–4.1 | 4.3–5.0 |
|  | Mean ± SD | 3.2 ± 0.2 | 3.8 ± 0.3 | 4.0 ± 0.4 | / | 3.3 ± 0.4 | 3.9 ± 0.3 | 4.7 ± 0.5 |
| UEW | Range | 2.7–3.2 | 3.0–4.3 | 2.7–4.4 | 3.5 | 2.5–3.9 | 2.5–2.8 | 2.7–3.1 |
|  | Mean ± SD | 3.0 ± 0.3 | 3.5 ± 0.5 | 3.5 ± 0.5 | / | 3.2 ± 0.3 | 2.6 ± 0.2 | 2.9 ± 0.3 |
| ED | Range | 4.7–4.9 | 4.7–6.2 | 4.5–5.5 | 5.4 | 4.3–5.7 | 5.0–5.7 | 5.6 |
|  | Mean ± SD | 4.8 ± 0.1 | 5.24± 0.5 | 5.0 ± 0.3 | / | 5.1 ± 0.4 | 5.3 ± 0.3 | 5.6 ± 0.0 |
| TYD | Range | 3.4–3.9 | 3.2–4.4 | 3.2–4.3 | 4.5 | 2.9–4.4 | 3.7–4.5 | 3.9–4.6 |
|  | Mean ± SD | 3.6 ± 0.3 | 3.7 ± 0.3 | 3.8 ± 0.4 | / | 3.3 ± 0.3 | 4.0 ± 0.4 | 4.3 ± 0.5 |
| LAL | Range | 18.1–20.1 | 20.5–26.1 | 18.6–22.6 | 24.9 | 8.6–10.8 | 8.9–20.7 | 10.4–22.5 |
|  | Mean ± SD | 19.2 ± 1.0 | 22.4 ± 1.8 | 21.0 ± 1.3 | / | 9.7 ± 0.6 | 14.8 ± 6.6 | 16.5 ± 8.6 |
| HAL | Range | 10.8–11.5 | 11.6–14.2 | 10.4–14.5 | 12.8 | 10.0–12.6 | 11.4–11.8 | 12.3–13.0 |
|  | Mean ± SD | 11.1 ± 0.4 | 12.6 ± 1.0 | 12.0 ± 1.1 | / | 11.1 ± 0.6 | 11.5 ± 0.2 | 12.7 ± 0.5 |
| LW | Range | 3.4–3.9 | 3.0–4.0 | 4.4–6.1 | 4.1 | 3.6–5.7 | 3.5–4.4 | 3.8 |
|  | Mean ± SD | 3.6 ± 0.3 | 3.5 ± 0.4 | 5.2 ± 0.5 | / | 4.6 ± 0.6 | 3.9 ± 0.4 | 3.8 ± 0.0 |
| THL | Range | 22.7–24.0 | 25.1–32.5 | 23.0–28.6 | 30.3 | 19.9–26.8 | 23.2–26.6 | 27.2–28.6 |
|  | Mean ± SD | 23.5 ± 0.7 | 28.2 ± 2.3 | 25.5 ± 1.7 | / | 23.2 ± 1.4 | 24.8 ± 1.4 | 27.9 ± 1.0 |
| TL | Range | 27.0–28.2 | 30.7–35.4 | 26.6–31.2 | 36.3 | 23.4–30.1 | 26.4–29.3 | 28.8–32.9 |
|  | Mean ± SD | 27.7 ± 0.6 | 32.5 ± 1.5 | 28.8 ± 1.7 | / | 26.0 ± 1.6 | 27.8 ± 1.2 | 30.9 ± 2.9 |
| TW | Range | 4.3–7.9 | 4.1–6.1 | 4.5–6.2 | 6.9 | 4.5–6.9 | 4.3–4.5 | 4.5–4.7 |
|  | Mean ± SD | 5.7 ± 1.9 | 5.0 ± 0.8 | 5.2 ± 0.6 | / | 5.3 ± 0.7 | 4.4 ± 0.08 | 4.6 ± 0.1 |
| TFL | Range | 35.7–36.4 | 37.7–45.5 | 33.9–40.4 | 42.8 | 31.0–39.5 | 34.8–38.0 | 40.0–44.4 |
|  | Mean ± SD | 36.0 ± 0.4 | 40.7 ± 2.8 | 36.8 ± 2.2 | / | 35.6 ± 2.0 | 36.8 ± 1.41 | 42.2 ± 3.1 |
| FL | Range | 24.2–25.7 | 24.6–30.7 | 23.4–27.5 | 29.5 | 21.2–27.1 | 24.1–26.4 | 28.4–31.5 |
|  | Mean ± SD | 25.2 ± 0.9 | 27.8 ± 2.2 | 25.4 ± 1.4 | / | 24.1 ± 1.4 | 25.4 ± 1.0 | 30.0 ± 2.2 |
| HDL/SVL | Range | 0.35–0.38 | 0.36–0.41 | 0.32–0.35 | 0.33 | 0.31–0.36 | 0.22–0.35 | 0.25–0.33 |
|  | Mean ± SD | 0.37 ± 0.02 | 0.37 ± 0.02 | 0.34 ± 0.01 | / | 0.33 ± 0.01 | 0.30 ± 0.06 | 0.29 ± 0.04 |
| HDW/HDL | Range | 0.78–0.86 | 0.81–0.93 | **0.93–1.03** | 0.94 | **0.91–1.08** | 0.85–1.46 | **0.97–1.26** |
|  | Mean ± SD | 0.83 ± 0.04 | 0.87 ± 0.04 | 0.98 ± 0.03 | / | 0.99 ± 0.04 | 1.1 ± 0.3 | 1.11 ± 0.21 |
| SL/SVL | Range | 0.14–0.16 | 0.14–0.16 | 0.13–0.17 | 0.13 | 0.12–0.15 | 0.13–0.16 | 0.14–0.15 |
|  | Mean ± SD | 0.15 ± 0.01 | 0.15 ± 0.01 | 0.15 ± 0.01 | / | 0.14 ± 0.01 | 0.15 ± 0.01 | 0.15 ± 0.01 |
| ED/HDL | Range | 0.28–0.31 | 0.25–0.30 | 0.30–0.36 | 0.30 | 0.32–0.38 | **0.34–0.53** | **0.36–0.44** |
|  | Mean ± SD | 0.29 ± 0.02 | 0.28 ± 0.01 | 0.33 ± 0.02 | / | 0.36 ± 0.02 | 0.43 ± 0.1 | 0.4 ± 0.06 |
| TYD/HDL | Range | 0.21–0.23 | 0.18–0.22 | 0.22–0.28 | 0.25 | 0.20–0.28 | **0.26–0.44** | **0.25–0.36** |
|  | Mean ± SD | 0.22 ± 0.01 | 0.20 ± 0.01 | 0.25 ± 0.02 | / | 0.23 ± 0.02 | 0.32 ± 0.08 | 0.3 ± 0.08 |
| TL/SVL | Range | 0.59–0.63 | 0.59–0.69 | 0.61–0.69 | 0.67 | 0.56–0.68 | 0.64–0.67 | 0.61–0.65 |
|  | Mean ± SD | 0.62 ± 0.02 | 0.64 ± 0.03 | 0.64 ± 0.02 | / | 0.61 ± 0.02 | 0.65 ± 0.02 | 0.63 ± 0.03 |
| THL/SVL | Range | 0.51–0.54 | 0.52–0.58 | 0.54–0.61 | 0.56 | 0.48–0.60 | **0.56–0.60** | 0.57 |
|  | Mean ± SD | 0.53 ± 0.02 | 0.55 ± 0.02 | 0.57 ± 0.02 | / | 0.54 ± 0.02 | 0.58 ± 0.02 | 0.57 ± 0 |
| TFL/SVL | Range | 0.80 | 0.71–0.85 | 0.77–0.87 | 0.79 | 0.76–0.89 | **0.82–0.91** | 0.84–0.88 |
|  | Mean ± SD | 0.80 ± 0 | 0.80 ± 0.04 | 0.82 ± 0.03 | / | 0.83 ± 0.03 | 0.86 ± 0.04 | 0.86 ± 0.03 |
| FL/SVL | Range | 0.54–0.58 | 0.49–0.59 | 0.54–0.59 | 0.54 | 0.52–0.61 | 0.58–0.62 | **0.60–0.63** |
|  | Mean ± SD | 0.56 ± 0.02 | 0.55 ± 0.03 | 0.57 ± 0.02 | / | 0.56 ± 0.03 | 0.59 ± 0.02 | 0.62 ± 0.02 |
| HAL/SVL | Range | 0.24–0.26 | 0.22–0.28 | 0.24–0.33 | 0.24 | 0.22–0.29 | 0.26–0.28 | 0.26 |
|  | Mean ± SD | 0.25 ± 0.01 | 0.25 ± 0.02 | 0.27 ± 0.02 | / | 0.26 ± 0.02 | 0.27 ± 0.01 | 0.26 ± 0 |
| LAL/SVL | Range | 0.41–0.44 | 0.38–0.48 | 0.44–0.49 | 0.46 | **0.21–0.25** | 0.22–0.49 | 0.22–0.45 |
|  | Mean ± SD | 0.43 ± 0.02 | 0.44 ± 0.03 | 0.47 ± 0.02 | / | 0.23 ± 0.01 | 0.34 ± 0.15 | 0.34 ± 0.16 |
